# Should Employers Be Permitted not to Hire Smokers? A Review of US Legal Provisions

**DOI:** 10.15171/ijhpm.2017.33

**Published:** 2017-03-15

**Authors:** Rishi R. Patel, Harald Schmidt

**Affiliations:** ^1^ College of Medicine, University of Kentucky, Lexington, KY, USA.; ^2^ Department of Medical Ethics and Health Policy, Perelman School of Medicine, University of Pennsylvania, Philadelphia, PA, USA.

**Keywords:** Tobacco Policy, Health Law, Ethics, Employment Discrimination, Denormalization

## Abstract

**Background:** Increasingly, healthcare and non-healthcare employers prohibit or penalize the use of tobacco products among current and new employees in the United States. Despite this trend, and for a range of different reasons, around half of states currently legally protect employees from being denied positions, or having employment contracts terminated, due to tobacco use.

**Methods:** We undertook a conceptual analysis of legal provisions in all 50 states.

**Results:** We found ethically relevant variations in terms of how tobacco is defined, which employee populations are protected, and to what extent they are protected. Furthermore, the underlying ethical rationales for smoker protection differ, and can be grouped into two main categories: prevention of discrimination and protection of privacy.

**Conclusion:** We critically discuss these rationales and the role of their advocates and argue that enabling equality of opportunity is a more adequate overarching concept for preventing employers from disadvantaging smokers.

## Introduction

Smoking causes an estimated 480 000 deaths annually in the United States and 5.7 million deaths globally, making it the most common cause of preventable mortality in the United States and worldwide.^1^ Smoking can also affect workplace productivity and is typically associated with higher healthcare cost. Should these facts play a role when it comes to employment decisions? Should we welcome non-hiring policies as a “tough-love” approach, in which we help smokers who want to quit but find it difficult to help themselves?^2,3^ In the United States, there is no federal law that would either prohibit employers from not hiring smokers, not one that would permit this in some cases. Instead, individual states legal frameworks guide these decisions. Increasingly, healthcare and non-healthcare employers are lawfully prohibiting employees from using tobacco.^4^ Contrary to this trend, and for a range of different reasons, approximately half of US states legally protect tobacco users in the employment context. What is legal is not necessarily ethical. Updating and expanding an earlier review on the subject,^5^ we analyze here legal rationales in favor and against hiring smokers and discuss their appropriateness from an ethical perspective. We suggest that the growing trend of legally disadvantaging tobacco users must be questioned, chiefly on grounds of fairness and equality of opportunity. Laws that bar employers from not hiring smokers should therefore be welcomed, even though the motivation and type of argument of those lobbying for such legislation must sometimes also be questioned.

## Methods

A comprehensive list of smoker protection legislation is maintained by Action on Smoking and Health (ASH).^6^ We ascertained that this list accurately reflected policies in effect as of January 2015 through supplementary literature research.^7^

We carried out content analysis of all identified laws and related legal documents including bill tracking summaries, bill histories, case descriptions, law reviews, and a governor’s address, using Lexis Advance® and LexisNexis® *Academic*. By utilizing open access through our respective university libraries, we searched the names and numbers of state statutes that were maintained by ASH and recorded any statutes that were listed and related to the particular topics. Along with typing in the statute names and numbers under the search drop bar, we individually searched the terms “tobacco,” smoking,” and “smoker” under the statutes tab for each state and Washington D.C. We obtained and analyzed a total of 61 documents between December 2014 and January 2015 (available from the authors).

### Limitations

We carried out a review and analysis of existing legal provisions concerning smokers hiring practices. The scope therefore does not extend to non-smoking policies in the workplace (resting on different ethical principles, as we note below), nor to actual employer practice and other aspects of implementation (such as which tests are used to determine smoking status and how accurate these are, or what share of employers, in practice, do not employ smokers—as opposed to offering free smoking cessation programs and then only refusing to employ smokers where they fail to quit provided with this support). While these and a range of further points are important and relevant to the most comprehensive assessment of the adequacy of non-hiring laws, our contribution here is focused specifically on the legal context, which, as will become clear, gives rise to an intricate set of issues all by itself.

## Results

Twenty-one states have no laws that protect tobacco users. The District of Columbia and 29 states have laws that offer some form of protection, including the leading tobacco-growing states of Kentucky and North Carolina.^8^ (These are: DC, CA, CO, CT, IL, IN, KY, LA, ME, MN, MS, MO, MT, NV, NH, NJ, NM, NY, NC, ND, OK, OR, RI, SC, SD, TN, VA, WV, WI, and WY: all abbreviations here and below refer to US postal notations for the US’ individual states.) As is shown in Supplementary file 1, the vast majority (28) of smoker protection laws were enacted between the late 1980s and early 1990s. We next describe the different ways in which using tobacco is referred to in these statutes, to whom the law applies, and the underlying rationales for protecting smokers as well as for exempting certain subgroups from protections.

### Broad and Narrow Descriptions of Tobacco-Use

The terminology used in laws has implications for what types of products are permitted or prohibited. Twenty states and one federal district use the terms “tobacco” or “tobacco products” in their laws (CT, DC, IN, KY, LA, ME, MN, MS, MO, MT, NH, NJ, NM, OK, OR, RI, SC, SD, VA, WV, and WY). Some states refer to “lawful products,” “lawful activities,” “lawful conduct,” or “lawful consumable products” (CA, CO, IL, MT, MN, NC, ND, and WI). New York refers to a “consumable product,” while Nevada and Tennessee focus on “the lawful use in this state of any product” and “the use of an agricultural product not regulated by the alcoholic beverage commission.”

### Rationales in Favor of Protecting Smokers

Rationales in support of protecting smokers can be clustered in two distinct categories: avoiding discrimination, and protecting privacy.

Seventeen states and Washington D.C. mention “discrimination” in the statutes even though none specifies the precise meaning of the term (LA, MT, NV, NH, NY, NC, WI, WV, WY, DC, IL, IN, KY, ME, MO, ND, RI, and SD). Washington D.C. and seven states cite the more narrowly constrained form of “employment discrimination” (DC, IL, IN, KY, ME,CT, NY, RI, WI, and SD).

Kentucky and North Dakota place smoking status alongside other fundamental civil rights categories. In Kentucky, employers may not:

*“Limit, segregate, or classify employees in any way which would deprive or tend to deprive an individual of employment opportunities or otherwise adversely affect status as an employee, because of the individual’s race, color, religion, national origin, sex, or age forty (40) and over, because the person is a qualified individual with a disability, or because the individual is a smoker or nonsmoker, as long as the person complies with any workplace policy concerning smoking. *”

Equally, In North Dakota,

“*[i]t is the policy of this state to prohibit discrimination on the basis of race, color, religion, sex, national origin, age, the presence of any mental or physical disability, status with regard to marriage or public assistance, or participation in lawful activity off the employer’s premises during nonworking hours which is not in direct conflict with the essential business-related interests of the employer.”*

The second rationale, found in 24 states, can be construed as being based on the overarching concept of privacy(CA, CO, CT, IL, MN, MS, MO, MT, NY, NC, ND, SD, WV, KY, LA, ME, NH, RI, VA, WY, OK, OR, TN, and WI). In a narrow sense, it entails that employers are permitted to require employees not to smoke at work, including during breaks, or while off site—even though employees may smoke outside of work hours altogether.

A broader sense of privacy is also found, and can be summarized as not permitting employees to smoke during working hours at the workplace, but allowing them to smoke during breaks on or off site, in addition to smoking when off duty.^9^ Four states permit the use of tobacco during “non-working hours” (OK, OR, TN, and WI).

### To Whom the Law Applies and Rationales in Support of Not Hiring Smokers

While the smoker protection policies reviewed here have the primary aim of enabling smokers to be hired (by preventing employers from not hiring them), they also include exceptions, and, as such, set out rationales in favor of not hiring. Only seven states cover all employees without exception [IN, KY, LA, ME, MS, NH, SC]. Statutes in 22 states and the District of Columbia include a range of nuanced distinctions for particular groups of employees (CA, CO, CT, DC, IL, MN, MO, MT, NV, NJ, NM, NC, ND, NY, OK, OR, RI, SD, TN, VA, WV, WI, and WY, see also Table 1 in Supplementary file 1, which lists specific job titles and further circumstances under which the laws are not applicable).

One prominent distinction is the exemption of employees who have *bona fide* occupational requirements or qualifications that they not smoke or use tobacco products. Eleven states use this language^4^ (CO, DC, MN, MT, NM, NC, OK, OR, SD, WI, and WY). Bona fide (literally: good faith) requirements typically require a rational basis and,^5^ for example, apply to employees who need to have a high level of physical fitness to be effective in fulfilling their professional obligations. Some states therefore expressly exclude police officers or firefighters from statutory protection (CA, CT, VA, SD, WI). In a similar vein, if the use of tobacco poses a safety or performance related concern to others, employees are exempt from the law in four states (NV, NC, MO, MT).

Another exemption is that some smoker protection policies do not apply to non-profit organizations, in which the use of tobacco products could be viewed as incompatible with the organizations’ overall purposes or objectives. Examples of such organizations range from cancer prevention groups to not for profit hospitals and religious organizations. Nine states permit non-hiring of smokers on these grounds (CO, CT, IL, MO, MT, RI, SD, WV, and WI).

In addition, New Jersey accepts non-hiring if a “rational basis” related to employment exists. North Dakota offers exemptions if there are direct conflicts with the “essential business-related interests of the employer.” Lastly, despite a *prima facie* prohibition on not hiring smokers, in Oregon, New York, Oklahoma, and Montana the policy is not applicable for collective bargaining agreements, nor is it applicable in Connecticut regarding such agreements covering paid firefighters or police officers.

Overall, the smoker protection policies differ regarding which types of employees are covered: most (17) extend to public employees, but 15 include private sector employees. They also differ regarding the kind of rationales that justify exemptions for not hiring or firing smokers. These comprised: if tobacco use adversely affects job performance or poses a safety concern, if it is incompatible with the purposes and objectives of a non-profit or religious organization, if there is a rational basis or essential business related interest, or if not smoking is part of a collective bargaining agreement.

## Discussion

### Variation Regarding the Use of Tobacco Products

Some smoker protection legislation refers to tobacco or the use of tobacco in a general manner such as consuming lawful or agricultural products, whereas other policies refer specifically to “tobacco” or “smoking.” Therefore, all states and Washington D.C. extend to the use of conventional cigarettes, but it is less clear whether electronic cigarettes would also be covered in 20 states that use the terms tobacco, tobacco products, or agricultural product (CT, DC, IN, KY, LA, ME, MS, MO, NH, NJ, NM, OK, OR, RI, SC, SD, VA, WV, WY, and TN). Nicotine may not be considered an agricultural product, insofar as it is not derived directly from tobacco but produced or isolated chemically.^10^ Broad construction permits flexibility in the statute, as producers of tobacco-related products bring to the market new goods over time that, as in the case of e-cigarettes, are viewed critically by the World Health Organization (WHO).^11^ The evidence on ethically relevant features of e-cigarettes such as their harmfulness, addictiveness and role in assisting with smoking cessation, or, conversely, taking up other forms of tobacco use is only just emerging.^12^ Whether or not arguments in favor or against protection of smokers of conventional tobacco products should, *mutatis mutandis*, apply to e-cigarettes therefore necessarily entails a considerable amount of speculation at this point. We bracket the case of e-cigarettes in what follows, but, insofar as the ethically relevant features should turn out to be largely identical to conventional tobacco products, would see no reason why the discussion that follows should not also apply here. On a more immediate and practical note, regulators will in any case need to review whether their definition of tobacco and or nicotine use will cover users of e-cigarettes or not.

### Variation Regarding the Type of Covered Employees

Smokers have different forms of protection in 29 states and Washington D.C., although 21 states have no protection whatsoever, raising the question of whether such variation should be acceptable. The same question arises in view of the noted difference in terms of covering both public and private employees, or public employees only (with a subset of the latter providing no exceptions for workers such as firemen or police officers). In addition there is variation in that none of the states that cover both public and private employees provide exceptions for non-profit or religious organizations in their statutes. We comment on how to respond to this variation after a discussion of the underlying rationales of protecting smokers.

### The Validity of Different Types of Rationales

Given the prominence of civil liberties-grounded rationales for protecting smokers, the variation in practice can be puzzling: either smokers in around half of states are deprived of fundamental rights,^6^ or the rationales are, in fact, less foundational than assumed by proponents.

Throughout the smoker protection legislation, 18 states and Washington D.C. draw on the concept of “discrimination” generally relying on employees to file civil actions or complaints with the government. Yet, smokers are not recognized as a protected class under federal law. While the argument has been made that the 1990 Americans with Disability Act—which may view alcoholics or recovering alcoholics as disabled—could be extended to tobacco,^13^ commentators^5^ and courts appear not persuaded as is also obvious from the fact that some 20 years on it is still legal to not employ smokers (whether through the absence of smoker protection laws, or through exemptions that such laws include). Moreover, even if tobacco users would be covered under the ADA, this would only apply to those who are addicted and not affect non-hiring occasional smokers. Overall, discrimination on the grounds of race, gender or disability—which would be based on largely immutable traits over which people have no, or only extremely limited control—are in a very different category, and rightly prohibited across all states universally.^14^ By contrast, no one is born a smoker. Nor is it clear that other conventionally required elements of direct and indirect forms of discrimination apply.^15^

Although smoker bans have clear moralistic overtones, a further and less overtly discussed reason for not hiring smokers is that they are presumed to have higher healthcare costs, and lower productivity.^2,9^ The argument has much currency in the private sector,^16^ yet is conspicuous in its absence in the legal documents reviewed here. It is not straightforward to quantify economic losses from smoking, which will vary greatly depending on the type of industry and extent of morbidity attributable to smoking. Estimates suggest that on average, incremental healthcare costs of around $2100 annually are associated with smoking (low range around $900, high range $3600). A further $3000 are attributed to excess absenteeism, smoking breaks, and lower productivity (low range around $2280, high range $4100).^4^ Clearly, noting such cost alone is insufficient as a reason for not employing smokers. But cost and the behavioral component do matter with regard to the question of the justification of smoker protection laws. Conceptually they complicate the relative ease of opposing non-hiring of smokers on grounds of discrimination: For while there are no adverse economic impacts, or risks to others, associated with some employees having, for example, a particular race or religion (paradigm cases of discrimination) such consequences are real in the case of tobacco at the population level.

The rationale of privacy, which was found in a broad and narrow version in 24 states, is a more complicated case. The widest defense of privacy would hold that smoking is as much a life-style choice that entails risks as is rock climbing, playing tennis, bicycling, or golfing: employees should therefore be free to pursue all these activities as part of their conception of what makes a life go well.^17^ One clear difference, is, however, that unlike these activities, smoking can be harmful to the health of coworkers, for example, where an employee smokes in close proximity to a coworker. It cannot be excluded that some employers therefore feel that the most effective form of protecting employees from secondary smoke is to simply not employ smokers. The harm principle, as initially set out in the 19th century by John Stuart Mill, has evolved to be a powerful public health norm and can support even severe limitations on individual liberty if another person is at risk of harm. For example, the harm principle can fully justify prohibiting smoking in public (or work) places—but precisely because these less intrusive, and more proportionate alternatives are available, it cannot be engaged to justify non-hiring. Employers may, of course, point to other forms of harm that can be attributed to smoking, chiefly, the above-cited higher healthcare costs, and lower productivity. Such cost may also arise from the cited sports. However, some activities also have health benefits; large proportions do not experience negative health effects; and the overall numbers and the collective impact on health budgets is far lower than in the case of smokers. As before, the cost argument alone cannot justify not hiring smokers, but arguments based on privacy also need to be seen in the broader context, which includes the economic impact.

An analysis of conceptual rationales must not ignore the role lobbyist and vested interest play. Many of the laws considered here have been initiated after active lobbying from the American Civil Liberties Union (ACLU), which typically focused on framing smoking as a private affair.^5,18^ In some instances, there has also been cooperation with tobacco industry lobby groups.^19^ For example, Philip Morris played a key role in creating New Hampshire’s smoker protection law in collaboration with the ACLU. The bill’s sponsor noted that a Philip Morris lobbyist, who also happened to be the former Speaker of the House, brought the bill forward.^10^ A collaboration can be surmised from a letter written by Lewis Maltby, an influential representative of the ACLU, to his coworker Jim Shields: “In 1988, Phillip Morris gave ACLU’s national office a three year grant for $75 000 per year to support our new initiative on civil liberties in the workplace…We have…worked closely with their public affairs department in support of particular legislation, and in discussing alternate national strategies.”^20^ Another, subsequently sent message further describes Mr. Maltby’s actions. Writing to Alan R. Miller, Philip Morris’s public affairs manager Maltby says: “I appreciate your offer to help us restructure our proposal…to help us resolve the $25 000 of last year’s funding which we never received.” Later that year, Crawford sent Maltby a letter enclosing a check “in support of ACLU…in…workplace discrimination ($100 000)…and…1993 activities ($25 000).”^21^ The timing of this support ties in with the increasing emergence of smoker protection legislation in the 1990s.

Avoiding discrimination and protecting privacy engages fundamental civil rights to enable smokers who want to smoke to continue to do so without incurring any disadvantage. But both lines of argument face the problems that smoking generates social and economic externalities. It is not clear how the fault lines should be reconciled. While it needs to be acknowledged that there are inevitable value trade offs, the problems that the discrimination and privacy rationales face do not mean that the case of protecting smokers needs to be abandoned. Rather, the focus should shift to a different rationale.

In our view, the most powerful rationale in favor of smoker protection legislation is the protection of fair employment opportunities, that is at least implicit in many of the statues that refer to discrimination, and in essence also captured in New Jersey’s statutes, that simply stipulate: “Smoking, or the use of tobacco products shall not affect employment.” This rationale is more appropriate and also easier to defend than those based on discrimination and privacy, as it can account for the specific circumstances in which people become smokers in the first place, which matter in ethical and legal terms.

If smoking were distributed equally across the population, fairness issues would take a different form. Yet, there are clear variations that underscore that not employing smokers disproportionately affects disadvantaged, vulnerable groups. First, almost half (around 45%) of the unemployed smoke, but only a third (or 28%) of those with full-time employment do so.^22^ Secondly, while more than 36% of those living below the federal poverty line are smokers, the rate decreases to 22.5% for those with incomes above the poverty level.^22^ Third, among adults with less than high school education, 32% are smokers; but among college graduates, smoking rates are just over 13%.^22^ Teenagers living in single parent households have higher levels of smoking than those with two parents.^23^ Not employing smokers hence hits those the hardest who are most in need of employment and have least opportunities to start with.

Proponents of not hiring smokers might accept these statistics, but argue that smokers could simply kick the habit^24^ and that tying employment to not smoking simply should be seen as offering a powerful incentive for behavior change.^2,3^ This would be required to tilt the balance in favor of any of the implicit and explicit ethical rationales that were identified in defense of hiring bans: higher healthcare costs and lower productivity, incompatibility of with the missions of employers engaged in healthcare delivery, research or policy, or essential business interests, all of which entail competing values. Equally, it would need to be accepted that smokers could stop smoking if one wanted to give priority to the further reasons which were found in some laws, that is, the existence of a rational basis, or a collective bargaining agreement.

However, such assumptions are unduly optimistic about the voluntariness of smoking. Nicotine is highly addictive, and tobacco use is not a free choice in the same way that one might choose between playing tennis rather than golf: approximately 69% of smokers want to quit,^8^ but only 3%-5% of initial unaided cessation attempts are successful.^25^ The overwhelming majority of smokers became addicted as teenagers, if not earlier.^22^ In comparison with alcohol use, only 3%-5% are alcoholics, demonstrating that alcohol, unlike nicotine, can be used by the majority of consumers in a non-addictive manner.^26^ Permitting not hiring of smokers also assumes that the policy is an effective tool for reducing smoking rates—yet it has been noted that there is a lack of published evidence on the claimed positive impact on population health.^27^ While the rationales in favor of not hiring smokers may seem justifiable to some, not protecting smokers risks increasing social inequality and stigmatization of vulnerable population groups.^27^ Non-hiring has also been questioned on purely economic grounds, advocating varying prices and wages as preferable alternatives.^28^ Lastly, in instituting hiring bans, employers (whether private or public, health or non-health sectors) abrogate a key public health responsibility. Instead of not hiring smokers and leaving them to help themselves (or, perhaps, offering free help with quitting smoking, but only hiring successful quitters) employers should make no difference in hiring and provide effective and evidence based smoking cessation services in the same way that they help other employees maintain their health, through preventative, curative, and rehabilitative services.

## Conclusion

Not hiring smokers threatens the social well-being and economic livelihood of many underprivileged people, in particular, those with low income, educational attainment and mental health problems. The practice therefore risks propagating social injustice and curbing social mobility with disproportionate impact on some of the most vulnerable members of society.

While some rationales cited in favor of non-hiring can be understandable, such as incompatibility with the missions of employers in the healthcare sector, and while a range of different trade-offs are inevitable, none can outweigh the cost of non-hiring across all types of professions. Laws that prohibit employers from not hiring smokers should therefore be adopted by all states. However, instead of basing these on preventing discrimination or protecting privacy, the strongest and most plausible rationale is to protect and enhance fair equality of opportunity. The only exception should be employees with genuine essential services requirements—such as police officers, firefighters or rescue workers with work profiles that require high levels of physical fitness—which can be justified based on the harm principle.^29^

## Acknowledgements

For most helpful extensive comments on earlier versions of the manuscript, we would like to thank Brenna Kelly. We are also grateful to Christopher Kuczynski for an important legal clarification, and to the three anonymous reviewers for enabling us to clarify central issues: as usual, any remaining errors and oversights are ours alone. We thank, Francis Terpening for editing assistance.

## Ethical issues

No particular ethics committee has approved this study. This study required no interaction with human participants. This paper includes an online-only annex that provides further detail on the legal status that were analysed. PDFs of all analysed documents are available from the authors.

## Competing interests

Authors declare that they have no competing interests.

## Authors’ contributions

RRP wrote the first draft of the manuscript and carried out the document acquisition and initial analysis. HS guided the study design and analysis and thoroughly reviewed and revised subsequent versions of the manuscript.

## Authors’ affiliations

^1^College of Medicine, University of Kentucky, Lexington, KY, USA. ^2^Department of Medical Ethics and Health Policy, Perelman School of Medicine, University of Pennsylvania, Philadelphia, PA, USA.

## Supplementary files

Supplementary file 1 Supplementary file 1 contains Table 1.

## Key messages

**Implications for policy makers**

- Curtailing employment opportunities for vulnerable populations by not hiring smokers is ethically unjustified, given that smoking is a medically recognized addiction, most smokers begin smoking under age, and smoking is distributed unequally across the population, with a larger share of worse off people, particularly unemployed populations: more efforts should therefore be made to ensure smokers are not disadvantaged.
- Due to definitional ambiguities relating to the term “tobacco use” and related concepts, it is not clear whether use of electronic cigarettes fall under the laws or not.
- With smoking becoming an increasing public health burden particularly in Africa and Asia, policy-makers in other countries should likewise take note and prioritize evidence based tobacco control and smoking cessation measures instead of adopting non-hiring practices.

**Implications for public**

When it comes to hiring and firing, should smokers be treated differently because smoking is a health risk? What does the law in each of the US’ 50 states say on the issue? These are the questions we examined, updating and expanding an earlier review on the subject. We found that a little over half of states protect smokers legally. A majority protects public employees, and a minority also extends to private employees. There are also noteworthy differences in the exemptions in smoker protection legislation: some states focus on job performance or safety, others on the purposes and objectives of the employer organization, as well as further categories. Finally, we identified two main overarching reasons that supported protecting smokers legally: to prevent discrimination, and to protect privacy. We consider the advantages and disadvantages of these rationales and propose that enabling equality of opportunity is a more adequate umbrella concept for preventing employers from disadvantaging smokers.
