# Supplementary material for: Should Employers Be Permitted not to Hire Smokers? A Review of US Legal Provisions
Source: Int J Health Policy Manag. 2017 Mar 15;6(12):701–6. doi: 10.15171/ijhpm.2017.33 (PMC5726320; doi:10.15171/ijhpm.2017.33)
Supplement: Supplementary file 1 — contains Table 1. [file ijhpm-6-701-s001.pdf]

Table 1. Smoker Protection Laws by State- Key Statures

This table reflects smoker protection laws as an effect of January 2016, in general, it does not separate out provisions included in the original act and/or enacted in subsequent revisions or amendments.

Name of state (* indicates tobacco growing state)	Name of principally relevant Law and Year Passed	Notes on Employee Groups exempted from protection	Rationales for smoker protection (verbatim citations from laws)	Percentage of smokers in state or district (2013)[1]
Illinois*	Right to Privacy in the Workplace Act<br><br>820 ILL. COMP. STAT. 55/5<br><br>1987	Employees who use tobacco products off duty during non-working hours or employees who are not part of a not for profit organization that has a purpose or objective to diminish tobacco use. Employees whose assigned duties are not impaired by their use of lawful products.	§ 5(a): "Except as otherwise specifically provided by law and except as provided in subsections (b) and (c) of this Section, it shall be unlawful for an employer to refuse to hire or to discharge any individual, or otherwise disadvantage any individual, with respect to compensation, terms, conditions or privileges of employment because the individual uses lawful products off the premises of the employer during nonworking hours."<br><br>§ 5(b): "This Section does not apply to any employer that is a non-profit organization that, as one of its primary purposes or objectives, discourages the use of one or more lawful products by the general public. This Section does not apply to the use of those lawful products which impairs an employee's ability to perform the employee's assigned duties."<br><br>§ 5(c): "It is not a violation of this Section for an employer to offer, impose or have in effect a health, disability or life insurance policy that makes distinctions between employees for the type of coverage or the price of coverage based upon the employees' use of lawful products provided that: (1) differential premium rates charged employees reflect a differential cost to the employer; and (2) employers provide employees with a statement delineating the differential rates used by insurance carriers."	21%
Oregon	OR. REV. STAT. § 659A.315 ("Use of tobacco during nonworking hours") & §659A.885 ("Civil Actions")<br><br>1989	Employees who do not have a *bona fide* requirement to not use tobacco or are not part of a collective bargaining agreement.	§ 659A.315. (1) It is an unlawful employment practice for any employer to require, as a condition of employment, that any employee or prospective employee refrain from using lawful tobacco products during nonworking hours, except when the restriction relates to a bona fide occupational requirement. (2) Subsection (1) of this section does not apply if an applicable collective bargaining agreement prohibits off-duty use of tobacco products.	18%

Virginia*	VA. CODE ANN. § 2.2-2902 (“Use of tobacco products by state employees”)<br><br>§ 27-40.1. Firefighters<br><br>§ 51.1-813. Police.<br><br>1989<br><br>§ 15.2-1504. (“Use of tobacco products by government employees”)<br><br>1997	All State and local governmental employees except firefighters and police officers.	2.2-2902. “No employee of or applicant for employment with the Commonwealth shall be required, as a condition of employment, to smoke or use tobacco products on the job, or to abstain from smoking or using tobacco products outside the course of his employment, provided that this section shall not apply to those classes of employees to which § 27-40.1 or § 51.1-813 is applicable.”<br><br>15.2-1504. “No employee of or applicant for employment with a locality or any political subdivision of the Commonwealth shall be required, as a condition of employment, to smoke or use tobacco products on the job, or to abstain from smoking or using tobacco products outside the course of his employment, provided that this section shall not apply to those classes of employees to which § 27-40.1 or § 51.1-813 are applicable.”	19%
Colorado	CO REV. STAT. ANN § 24-34-402.5 (“Unlawful prohibition of legal activities as a condition of employment”)<br><br>1990	The prohibition on smoking during nonworking hours is permissible if the employees have a *bona fide* requirement to not use tobacco or if the prohibition is reasonably and rationally related to employment	(1) It shall be a discriminatory or unfair employment practice for an employer to terminate the employment of any employee due to that employee's engaging in any lawful activity off the premises of the employer during nonworking hours unless such a restriction:<br>(a) Relates to a bona fide occupational requirement or is reasonably and rationally related to the employment activities and responsibilities of a particular employee or a particular group of employees, rather than to all employees of the employer; or<br>(b) Is necessary to avoid a conflict of interest with any responsibilities to the employer or the appearance of such a conflict of interest.<br>(2)(a) Notwithstanding any other provisions of this article, the sole remedy for any person claiming to be aggrieved by a discriminatory or unfair employment practice as defined in this section shall be as follows: He or she may bring a civil action for damages in any district court of competent jurisdiction and may sue for all wages and benefits that would have been due him or her up to and including the date of the judgment had the discriminatory or unfair employment practice not occurred; except that nothing in this section shall be construed to relieve the person from the obligation to mitigate his or her damages.	20%

		activities or responsibilities. Employees who work for a not for profit organization in which the use of tobacco would produce a conflict of interest may also not be protected and those who work for an employer who employs fewer than 15 people are not protected.	(b)(I) If the prevailing party in the civil action is the plaintiff, the court shall award the plaintiff court costs and a reasonable attorney fee.<br><br>(II) This paragraph (b) shall not apply to an employee of a business that has or had fifteen or fewer employees during each of twenty or more calendar work weeks in the current or preceding calendar year.	
South Carolina*	S.C. CODE ANN. § 41-1-85 (“Personnel action based on use of tobacco products outside of workplace prohibited”)<br><br>1990	All employees	“The use of tobacco products outside the workplace must not be the basis of personnel action, including, but not limited to, employment, termination, demotion, or promotion of an employee.”	24%
Tennessee*	TENN. CODE ANN. § 50-1-304<br><br>1990		(d)(1) No employee shall be discharged or terminated solely for participating or engaging in the use of an agricultural product not regulated by the alcoholic beverage commission that is not otherwise proscribed by law, if the employee participates or engages in the use in a manner that complies with all applicable employer policies regarding the use during times at which the employee is working.<br>(2) No employee shall be discharged or terminated solely for participating or engaging in the use of the product not regulated by the alcoholic beverage commission that is not otherwise proscribed by law if the employee participates or engages in the activity during times when the employee is not working.	25%
Louisiana	LA REV. STAT. ANN. § 23:966 (“Prohibition of smoking discrimination”)<br>1991	All Employees	A. As long as an individual, during the course of employment, complies with applicable law and any adopted workplace policy regulating smoking, it shall be unlawful for an employer:<br>(1) To discriminate against the individual with respect to discharge, compensation, promotion, any personnel action or other condition, or privilege of employment because the individual is a smoker or nonsmoker.<br>(2) To require, as a condition of employment, that the individual abstain from smoking or otherwise using tobacco products outside the course of employment.	26%

			B. A smoker, as referred to herein, is limited to a person who smokes tobacco.<br>C. Nothing in this Section shall preclude an employer from formulating and adopting a policy regulating an employee's workplace use of a tobacco product or from taking any action consistent therewith.<br>D. Any employer who violates the provisions of this Section shall be fined up to two hundred fifty dollars for the first offense and up to five hundred dollars for any subsequent offense.	
Maine	ME REV. STAT. ANN. tit. 26, § 597<br><br>(“Conditions of Employment”)<br><br>1991	All Employees	An employer or an agent of an employer may not require, as a condition of employment, that any employee or prospective employee refrain from using tobacco products outside the course of that employment or otherwise discriminate against any person with respect to the person's compensation, terms, conditions or privileges of employment for using tobacco products outside the course of employment as long as the employee complies with any workplace policy concerning use of tobacco.	20%
Nevada	NEV. REV. STAT. § 613.333 (“Unlawful employment practices: Discrimination for lawful use of any product outside premises of employer which does not adversely affect job performance or safety of other employee”)<br><br>1991	Employees in which tobacco does not adversely affect job performance or safety of other employees	1. It is an unlawful employment practice for an employer to:<br>(a) Fail or refuse to hire a prospective employee; or<br>(b) Discharge or otherwise discriminate against any employee concerning the employee’s compensation, terms, conditions or privileges of employment, because the employee engages in the lawful use in this state of any product outside the premises of the employer during the employee’s nonworking hours, if that use does not adversely affect the employee’s ability to perform his or her job or the safety of other employees.<br>2. An employee who is discharged or otherwise discriminated against in violation of subsection 1 or a prospective employee who is denied employment because of a violation of subsection 1 may bring a civil action against the employer who violates the provisions of subsection 1 and obtain:<br>(a) Any wages and benefits lost as a result of the violation;<br>(b) An order of reinstatement without loss of position, seniority or benefits;<br>(c) An order directing the employer to offer employment to the prospective employee; and<br>(d) Damages equal to the amount of the lost wages and benefits.<br>3. The court shall award reasonable costs, including court costs and attorney's fees to the prevailing party in an action brought pursuant to this section.<br>4. The remedy provided for in this section is the exclusive remedy for an action brought pursuant to this section.	24%
New Hampshire	N.H. REV. STAT. ANN. § 275:37-a ("Discrimination on Basis of Using Tobacco Products Prohibited”)<br><br>1991	All employees	No employer shall require as a condition of employment that any employee or applicant for employment abstain from using tobacco products outside the course of employment, as long as the employee complies with any workplace policy….	17%

New Jersey	N.J. STAT. ANN. § 34:6B-1 ("Employer discrimination against persons who do or do not smoke or use tobacco products prohibited")<br><br>1991	Does not apply to employees if a rational basis which is reasonably related to the employment, including the responsibilities of the employee.	No employer shall refuse to hire or employ any person or shall discharge from employment or take any adverse action against any employee with respect to compensation, terms, conditions or other privileges of employment because that person does or does not smoke or use other tobacco products, unless the employer has a rational basis for doing so which is reasonably related to the employment, including the responsibilities of the employee or prospective employee.	18%
New Mexico	N.M. STAT. ANN. § 50-11-3 ("Employers; unlawful practices")<br><br>N.M. STAT. ANN. § 50-11-4. ("Remedies") 1991	Does not apply if it materially threatens an employer's legitimate conflict of interest policy reasonably designed to protect the employer's proprietary interests or produces a *bona fide* requirement that is reasonably or rationally related.	A. It is unlawful for an employer to:<br>(1) Refuse to hire or to discharge any individual, or otherwise disadvantage any individual, with respect to compensation, terms, conditions or privileges of employment because the individual is a smoker or nonsmoker, provided that the individual complies with applicable laws or policies regulating smoking on the premises of the employer during working hours; or<br>(2) Require as a condition of employment that any employee or applicant for employment abstain from smoking or using tobacco products during nonworking hours, provided the individual complies with applicable laws or policies regulating smoking on the premises of the employer during working hours.<br>B. The provisions of Subsection A of this section shall not be deemed to protect any activity that:<br>(1) Materially threatens an employer's legitimate conflict of interest policy reasonably designed to protect the employer's trade secrets, proprietary information or other proprietary interests; or<br>(2) Relates to a bona fide occupational requirement and is reasonably and rationally related to the employment activities and responsibilities of a particular employee or a particular group of employees, rather than to all employees of the employer.	20%
North Carolina*	N.C. GEN. STAT. § 95-28.2. ("Discrimination against persons for lawful use of lawful products during nonworking hours prohibited")	Employees who do not have a *bona fide* requirement to not use tobacco that is reasonably related to the employment activities.<br>The law does not apply to employers who employ less than	(a) As used in this section, "employer" means the State and all political subdivisions of the State, public and quasi-public corporations, boards, bureaus, commissions, councils, and private employers with three or more regularly employed employees.<br>(b) It is an unlawful employment practice for an employer to fail or refuse to hire a prospective employee, or discharge or otherwise discriminate against any employee with respect to compensation, terms, conditions, or privileges of employment because the prospective employee or the employee engages in or has engaged in the lawful use of lawful products if the activity occurs off the premises of the employer during nonworking hours and does not adversely affect the employee's job performance or the person's ability to properly fulfill the responsibilities of the position in question or the safety of other employees.<br>(c) It is not a violation of this section for an employer to do any of the following:<br>(1) Restrict the lawful use of lawful products by employees during nonworking hours if the restriction relates to a bona fide occupational requirement and is reasonably related to the employment activities. If the restriction reasonably relates to only a particular employee or group of employees, then the restriction may only lawfully apply to them.	23%

	1991	three employees. The protection does not apply if the restriction imposed relates to the fundamental objectives of the organization.	(2) Restrict the lawful use of lawful products by employees during nonworking hours if the restriction relates to the fundamental objectives of the organization.<br>(3) Discharge, discipline, or take any action against an employee because of the employee's failure to comply with the requirements of the employer's substance abuse prevention program or the recommendations of substance abuse prevention counselors employed or retained by the employer.<br>(d) This section shall not prohibit an employer from offering, imposing, or having in effect a health, disability, or life insurance policy distinguishing between employees for the type or price of coverage based on the use or nonuse of lawful products if each of the following is met:<br>(1) Differential rates assessed employees reflect actuarially justified differences in the provision of employee benefits.<br>(2) The employer provides written notice to employees setting forth the differential rates imposed by insurance carriers.<br>(3) The employer contributes an equal amount to the insurance carrier on behalf of each employee of the employer.<br>(e) An employee who is discharged or otherwise discriminated against, or a prospective employee who is denied employment in violation of this section, may bring a civil action within one year from the date of the alleged violation against the employer who violates the provisions of subsection (b) of this section and obtain any of the following:<br>(1) Any wages or benefits lost as a result of the violation;<br>(2) An order of reinstatement without loss of position, seniority, or benefits; or<br>(3) An order directing the employer to offer employment to the prospective employee.<br><br>(f) The court may award reasonable costs, including court costs and attorneys' fees, to the prevailing party in an action brought pursuant to this section.	
Oklahoma	40 OKLA. STAT. ANN. § 500 (“Nonsmoking as condition of employment”)<br><br>40 Okl.St.Ann. § 502 (“Application of act--Restriction on smoking as bona fide occupational requirement or part of collective bargaining agreement”)<br><br>1991	Employers may restrict non-working smoking when the restriction on smoking relates to a bona fide occupational requirement or an applicable collective bargaining agreement which prohibits or allows off-duty use of tobacco products.	§ 501. A. It shall be unlawful for an employer to:<br>1. Discharge any individual, or otherwise disadvantage any individual, with respect to compensation, terms, conditions or privileges of employment because the individual is a nonsmoker or smokes or uses tobacco products during nonworking hours; or<br>2. Require as a condition of employment that any employee or applicant for employment abstain from smoking or using tobacco products during nonworking hours.<br>B. Nothing in this section shall prohibit an employer from offering incentives to an employee to participate in wellness programs, including, but not limited to, smoking cessation programs, in conjunction with the employer providing the employee health insurance coverage.<br><br>§ 502. The provisions of Sections 11 through 14 of this act (Sections 500 to 503) shall not apply when the restriction on smoking relates to a bona fide occupational requirement or an applicable collective bargaining agreement which prohibits or allows off-duty use of tobacco products.	26%

South Dakota	S.D. CODIFIED LAWS § 60-4-11 (“Discrimination against employee off-duty use of tobacco”) 1991	Employees who do not have a *bona fide* requirement to not use tobacco and employees who do not work for a not for profit organization in which the use of tobacco would produce a conflict of interest.<br><br>Firefighters are not protected by this statute.	It is a discriminatory or unfair employment practice for an employer to terminate the employment of an employee due to that employee's engaging in any use of tobacco products off the premises of the employer during nonworking hours unless such a restriction:<br>(1) Relates to a bona fide occupational requirement and is reasonably and rationally related to the employment activities and responsibilities of a particular employee or a particular group of employees, rather than to all employees of the employer; or<br>(2) Is necessary to avoid a conflict of interest with any responsibilities to the employer or the appearance of such a conflict of interest.<br><br>Notwithstanding any other provisions of this chapter, the sole remedy for any person claiming to be aggrieved by a discriminatory or unfair employment practice as defined in this section shall be as follows: the person may bring a civil suit for damages in circuit court and may sue for all wages and benefits which have been due up to and including the date of the judgment had the discriminatory or unfair employment practice not occurred. However, nothing in this section may be construed to relieve such person from the obligation to mitigate damages. It is not a discriminatory or unfair employment practice pursuant to this section for an employer to offer, impose or have in effect a health or life insurance policy that makes distinctions between employees for the type of coverage or the cost of coverage based upon the employees' use of tobacco products. The provisions of this section shall not apply to full-time firefighters.	22%
Wisconsin*	WIS. STAT. §§ 111.31 (“Declaration of policy”) and 111.35 (“Use or nonuse of lawful products; exceptions and special cases”) 1991	Employees whose use of the lawful product impairs the employee’s ability to adequately perform job-related responsibilities, creates a conflict of interest or the appearance of a conflict of interest, or is a *bona fide* requirement that is reasonably related to the employee’s job-related responsibilities. Employees who do not work for a not for profit organization in which one of its primary	111.35 (1)(a) Notwithstanding s. 111.322, it is not employment discrimination because of use of a lawful product off the employer's premises during nonworking hours for a nonprofit corporation that, as one of its primary purposes or objectives, discourages the general public from using a lawful product to refuse to hire or employ an individual, to suspend or terminate the employment of an individual, or to discriminate against an individual in promotion, in compensation or in terms, conditions or privileges of employment, because that individual uses off the employer's premises during nonworking hours a lawful product that the nonprofit corporation discourages the general public from using.<br>(b) Notwithstanding s. 111.322, it is not employment discrimination because of nonuse of a lawful product off the employer's premises during nonworking hours for a nonprofit corporation that, as one of its primary purposes or objectives, encourages the general public to use a lawful product to refuse to hire or employ an individual, to suspend or terminate the employment of an individual, or to discriminate against an individual in promotion, in compensation or in terms, conditions or privileges of employment, because that individual does not use off the employer's premises during nonworking hours a lawful product that the nonprofit corporation encourages the general public to use.<br>(2) Notwithstanding s. 111.322, it is not employment discrimination because of use or nonuse of a lawful product off the employer's premises during nonworking hours for an employer, labor organization, employment agency, licensing agency or other person to refuse to hire, employ, admit, or license an individual, to bar, suspend or terminate an individual from employment, membership or licensure, or to discriminate against an individual in promotion, in compensation or in terms, conditions or privileges of employment or labor organization membership if the individual's use or nonuse of a lawful product off the employer's premises during nonworking hours does any of the following:<br>(a) Impairs the individual's ability to undertake adequately the job-related responsibilities of that individual's employment, membership or licensure.<br>(b) Creates a conflict of interest, or the appearance of a conflict of interest, with the job-related responsibilities of that individual's employment, membership or licensure.<br>(c) Conflicts with a bona fide occupational qualification that is reasonably related to the job-related responsibilities of that individual's employment, membership or licensure.<br>(d) Constitutes a violation of s. 254.92(2).	20%

		objectives or purposes is to discourage the general public from using a lawful product State, county and municipal firefighters are not covered.	(e) Conflicts with any federal or state statute, rule or regulation.<br>(3)(a) Notwithstanding s. 111.322, it is not employment discrimination because of use of a lawful product off the employer's premises during nonworking hours for an employer, labor organization, employment agency, licensing agency or other person to offer a policy or plan of life, health or disability insurance coverage under which the type of coverage or the price of coverage for an individual who uses a lawful product off the employer's premises during nonworking hours differs from the type of coverage or the price of coverage provided for an individual who does not use that lawful product, if all of the following conditions apply:<br>1. The difference between the premium rates charged to an individual who uses that lawful product and the premium rates charged to an individual who does not use that lawful product reflects the cost of providing the coverage to the individual who uses that lawful product.<br>2. The employer, labor organization, employment agency, licensing agency or other person that offers the coverage provides each individual who is charged a different premium rate based on that individual's use of a lawful product off the employer's premises during nonworking hours with a written statement specifying the premium rate differential used by the insurance carrier.<br>(b) Notwithstanding s. 111.322, it is not employment discrimination because of nonuse of a lawful product off the employer's premises during nonworking hours for an employer, labor organization, employment agency, licensing agency or other person to offer a policy or plan of life, health or disability insurance coverage under which the type of coverage or the price of coverage for an individual who does not use a lawful product off the employer's premises during nonworking hours differs from the type of coverage or the price of coverage provided for an individual who uses that lawful product, if all of the following conditions apply:<br>1. The difference between the premium rates charged to an individual who does not use that lawful product and the premium rates charged to an individual who uses that lawful product reflects the cost of providing the coverage to the individual who does not use that lawful product.<br>2. The employer, labor organization, employment agency, licensing agency or other person that offers the coverage provides each individual who is charged a different premium rate based on that individual's nonuse of a lawful product off the employer's premises during nonworking hours with a written statement specifying the premium rate differential used by the insurance carrier.<br>(4) Notwithstanding s. 111.322, it is not employment discrimination because of use of a lawful product off the employer's premises during nonworking hours to refuse to employ an applicant if the applicant's use of a lawful product consists of smoking tobacco and the employment is as a fire fighter covered under s. 891.45 or 891.455.	
Minnesota	MINN. STAT. § 181.938 (“Nonwork activities; prohibited employer conduct”)<br><br>1992	Employees who do not have a *bona fide* requirement to not use tobacco and employees who do not work for an organization in which the use of tobacco would produce a conflict of interest.	**Subdivision 1. Definition.** For the purpose of this section, “employer” has the meaning given it in section 179.01, subdivision 3.<br>**Subd. 2. Prohibited practice.** An employer may not refuse to hire a job applicant or discipline or discharge an employee because the applicant or employee engages in or has engaged in the use or enjoyment of lawful consumable products, if the use or enjoyment takes place off the premises of the employer during nonworking hours. For purposes of this section, “lawful consumable products” means products whose use or enjoyment is lawful and which are consumed during use or enjoyment, and includes food, alcoholic or nonalcoholic beverages, and tobacco.<br>**Subd. 3. Exceptions.** (a) It is not a violation of subdivision 2 for an employer to restrict the use of lawful consumable products by employees during nonworking hours if the employer's restriction:<br>(1) Relates to a bona fide occupational requirement and is reasonably related to employment activities or responsibilities of a particular employee or group of employees; or<br>(2) Is necessary to avoid a conflict of interest or the appearance of a conflict of interest with any responsibilities owed by the employee to the employer.	17%

		The statute does not generally protect state employees.	(b) It is not a violation of subdivision 2 for an employer to refuse to hire an applicant or discipline or discharge an employee who refuses or fails to comply with the conditions established by a chemical dependency treatment or aftercare program.<br>(c) It is not a violation of subdivision 2 for an employer to offer, impose, or have in effect a health or life insurance plan that makes distinctions between employees for the type of coverage or the cost of coverage based upon the employee's use of lawful consumable products, provided that, to the extent that different premium rates are charged to the employees, those rates must reflect the actual differential cost to the employer.<br>(d) It is not a violation of subdivision 2 for an employer to refuse to hire an applicant or discipline or discharge an employee on the basis of the applicant's or employee's past or present job performance.<br><br>**Subd. 4. Remedy.** The sole remedy for a violation of subdivision 2 is a civil action for damages. Damages are limited to wages and benefits lost by the individual because of the violation. A court shall award the prevailing party in the action, whether plaintiff or defendant, court costs and a reasonable attorney fee.	
Missouri*	MO. REV. STAT. § 290.145 (“Discrimination, refusal to hire or discharge employee for alcohol or tobacco use not during working hours, prohibited, exception--not cause for legal actions”)<br><br>1992	Does not apply to religious organizations, church-operated institutions, and not for profit organizations in which tobacco use would produce a conflict of interest.<br><br>Does not apply to employees whose use of alcohol or tobacco during nonwork hours interferes with the duties and performance of the employee, the employee's coworkers, or the overall operation of the employer's business.	It shall be an improper employment practice for an employer to refuse to hire, or to discharge, any individual, or to otherwise disadvantage any individual, with respect to compensation, terms or conditions of employment because the individual uses lawful alcohol or tobacco products off the premises of the employer during hours such individual is not working for the employer, unless such use interferes with the duties and performance of the employee, the employee's coworkers, or the overall operation of the employer's business; except that, nothing in this section shall prohibit an employer from providing or contracting for health insurance benefits at a reduced premium rate or at a reduced deductible level for employees who do not smoke or use tobacco products. Religious organizations and church-operated institutions, and not-for-profit organizations whose principal business is health care promotion shall be exempt from the provisions of this section. The provisions of this section shall not be deemed to create a cause of action for injunctive relief, damages or other relief.	26%
New York	N.Y. [LABOR] LAW § 201-d (“Discrimination against the	The protection does not apply to when the	1. Definitions. As used in this section:	19%

	engagement in certain activities")<br><br>1992	employer's legal use of consumable products creates a material conflict of interest related to the employer's trade secrets, proprietary information or other proprietary or business interest.<br><br>There are likely more exceptions, but this law is complicated, as it cross-references several other laws, and, as such, will take a bit more time to research. It looks like those subject to collective bargaining agreement and some state / city employees may be exempted from this protection as well.	a. "Political activities" shall mean (i) running for public office, (ii) campaigning for a candidate for public office, or (iii) participating in fund-raising activities for the benefit of a candidate, political party or political advocacy group;<br>b. "Recreational activities" shall mean any lawful, leisure-time activity, for which the employee receives no compensation and which is generally engaged in for recreational purposes, including but not limited to sports, games, hobbies, exercise, reading and the viewing of television, movies and similar material;<br>c. "Work hours" shall mean, for purposes of this section, all time, including paid and unpaid breaks and meal periods, that the employee is suffered, permitted or expected to be engaged in work, and all time the employee is actually engaged in work. This definition shall not be referred to in determining hours worked for which an employee is entitled to compensation under any law including article nineteen of this chapter.<br>2. Unless otherwise provided by law, it shall be unlawful for any employer or employment agency to refuse to hire, employ or license, or to discharge from employment or otherwise discriminate against an individual in compensation, promotion or terms, conditions or privileges of employment because of:<br>a. An individual's political activities outside of working hours, off of the employer's premises and without use of the employer's equipment or other property, if such activities are legal, provided, however, that this paragraph shall not apply to persons whose employment is defined in paragraph six of subdivision (a) of section seventy-nine-h of the civil rights law, and provided further that this paragraph shall not apply to persons who would otherwise be prohibited from engaging in political activity pursuant to chapter 15 of title 5 and subchapter III of chapter 73 of title 5 of the USCA;<br>b. An individual's legal use of consumable products prior to the beginning or after the conclusion of the employee's work hours, and off of the employer's premises and without use of the employer's equipment or other property;<br>c. An individual's legal recreational activities outside work hours, off of the employer's premises and without use of the employer's equipment or other property; or<br>d. An individual's membership in a union or any exercise of rights granted under Title 29, USCA, Chapter 7 or under article fourteen of the civil service law.<br>3. The provisions of subdivision two of this section shall not be deemed to protect activity which:<br>a. Creates a material conflict of interest related to the employer's trade secrets, proprietary information or other proprietary or business interest;<br>b. With respect to employees of a state agency as defined in sections seventy-three and seventy-four of the public officers law respectively, is in knowing violation of subdivision two, three, four, five, seven, eight or twelve of section seventy-three or of section seventy-four of the public officers law, or of any executive order, policy, directive, or other rule which has been issued by the attorney general regulating outside employment or activities that could conflict with employees' performance of their official duties;<br>c. With respect to employees of any employer as defined in section twenty-seven-a of this chapter, is in knowing violation of a pro-vision of a collective bargaining agreement concerning ethics, conflicts of interest, potential conflicts of interest, or the proper dis-charge of official duties;<br>d. With respect to employees of any employer as defined in section twenty-seven-a of this chapter who are not subject to section seventy-three or seventy-four of the public officers law, is in knowing violation of article eighteen of the general municipal law or any local law, administrative code provision, charter provision or rule or directive of the mayor or any agency head of a city having a population of one million or more, where such law, code provision, charter provision, rule or directive concerns ethics, conflicts of interest, potential conflicts of interest, or the proper discharge of official duties and otherwise covers such employees; and<br>e. With respect to employees other than those of any employer as defined in section twenty-seven-a of this chapter, violates a collective bargaining agreement or a certified or licensed professional's

			contractual obligation to devote his or her entire compensated working hours to a single employer, provided however that the provisions of this paragraph shall apply only to professionals whose compensation is at least fifty thousand dollars for the year nineteen hundred ninety-two and in subsequent years is an equivalent amount adjusted by the same percentage as the annual increase or decrease in the consumer price index.<br>4. Notwithstanding the provisions of subdivision three of this section, an employer shall not be in violation of this section where the employer takes action based on the belief either that: (i) the employer's actions were required by statute, regulation, ordinance or other governmental mandate, (ii) the employer's actions were permissible pursuant to an established substance abuse or alcohol program or workplace policy, professional contract or collective bargaining agreement, or (iii) the individual's actions were deemed by an employer or previous employer to be illegal or to constitute habitually poor performance, incompetency or misconduct.<br>5. Nothing in this section shall apply to persons who, on an individual basis, have a professional service contract with an employer and the unique nature of the services provided is such that the employer shall be permitted, as part of such professional service contract, to limit the off-duty activities which may be engaged in by such individual.<br>6. Nothing in this section shall prohibit an organization or employer from offering, imposing or having in effect a health, disability or life insurance policy that makes distinctions between employees for the type of coverage or the price of coverage based upon the employees' recreational activities or use of consumable products, provided that differential premium rates charged employees reflect a differential cost to the employer and that employers provide employees with a statement delineating the differential rates used by the carriers providing insurance for the employer, and provided further that such distinctions in type or price of coverage shall not be utilized to expand, limit or curtail the rights or liabilities of any party with regard to a civil cause of action.<br>7. a. Where a violation of this section is alleged to have occurred, the attorney general may apply in the name of the people of the state of New York for an order enjoining or restraining the commission or continuance of the alleged unlawful acts. In any such proceeding, the court may impose a civil penalty in the amount of three hundred dollars for the first violation and five hundred dollars for each subsequent violation.<br>b. In addition to any other penalties or actions otherwise applicable pursuant to this chapter, where a violation of this section is alleged to have occurred, an aggrieved individual may commence an action for equitable relief and damages.	
West Virginia*	W. VA. CODE § 21-3-19 (“Discrimination for use of tobacco products prohibited”)<br><br>1992	Employees who do not work for a not for profit organization which, as one of its primary purposes, discourages the use of one or more tobacco products by the general public.	(a) It shall be unlawful for any employer, whether public or private, or the agent of such employer to refuse to hire any individual or to discharge any employee or otherwise to disadvantage or penalize any employee with respect to compensation, terms, conditions or privileges of employment solely because such individual uses tobacco products off the premises of the employer during nonworking hours.<br>(b) This section shall not apply with respect to an employer which is a nonprofit organization which, as one of its primary purposes or objectives, discourages the use of one or more tobacco products by the general public.<br>(c) This section shall not prohibit an employer from offering, imposing or having in effect a health, disability or life insurance policy which makes distinctions between employees for type of coverage or price of coverage based upon the employee's use of tobacco products: Provided, That any differential premium rates charged to employees must reflect differential costs to the employer: Provided, however, That the employer must provide employees with a statement delineating the differential rates used by its insurance carriers.	25%

			(d) Nothing in this section shall be construed to prohibit an employer from making available to smokers and other users of tobacco products, programs, free of charge or at reduced rates, which encourage the reduction or cessation of smoking or tobacco use.	
Wyoming	WYO. STAT. ANN. § 27-9-105 (“Discriminatory and unfair employment practices enumerated; limitation”) 1992	Employees who do not have a *bona fide* occupational qualification to not use tobacco	(a)(iv) For an employer to require as a condition of employment that any employee or prospective employee use or refrain from using tobacco products outside the course of his employment, or otherwise to discriminate against any person in matters of compensation or the terms, conditions or privileges of employment on the basis of use or nonuse of tobacco products outside the course of his employment unless it is a bona fide occupational qualification that a person not use tobacco products outside the workplace. Nothing within this paragraph shall prohibit an employer from offering, imposing or having in effect a health, disability or life insurance policy distinguishing between employees for type or price of coverage based upon the use or nonuse of tobacco products if:<br>(A) Differential rates assessed employees reflect an actual differential cost to the employer; and<br>(B) Employers provide written notice to employees setting forth the differential rates imposed by insurance carriers.	21%
Washington D.C.	D.C. CODE ANN. § 7-1703.03 (“Prohibition of employment discrimination on the basis of tobacco use”)<br><br>1993	The protection does not apply to employees whose employers establish that tobacco-use restrictions or prohibitions constitute bona fide occupational qualifications.	(a) No person shall refuse to hire or employ any applicant for employment, or discharge or otherwise discriminate against any employee with respect to compensation or any other term, condition, or privilege of employment, on the basis of the use by the applicant or employee of tobacco or tobacco products. Nothing in this section shall be construed as limiting a person from establishing or enforcing workplace smoking restrictions that are required or permitted by this subchapter or other District or federal laws, or in establishing tobacco-use restrictions or prohibitions that constitute bona fide occupational qualifications.<br>(b) Any employee or applicant for employment who is aggrieved by a violation of subsection (a) of this section shall have a private cause of action against the person. An employee or applicant for employment shall pursue and exhaust all remedies available pursuant to any collective bargaining agreement, grievance procedure, or other established means of resolving employer-employee disputes, to resolve a violation of subsection (a) of this section, prior to commencing a civil action.<br>(c) Any employee or applicant for employment who is aggrieved by a violation of subsection (a) of this section shall be entitled to recover any damages, including lost or back wages or salary. The court, in its discretion, may allow the prevailing party a reasonable attorney's fee as part of the costs.	19%
Montana	MONT. CODE ANN. §§ 39-2-313 & 39-2-314<br><br>1993	Employees where their use of tobacco affects in any manner an individual's ability to perform job-related employment responsibilities or the safety of other employees. Does not apply to certain employees with professional service contracts or to non-profit	39-2-313. (1) For purposes of this section, “lawful product” means a product that is legally consumed, used, or enjoyed and includes food, beverages, and tobacco.<br>(2) Except as provided in subsections (3) and (4), an employer may not refuse to employ or license and may not discriminate against an individual with respect to compensation, promotion, or the terms, conditions, or privileges of employment because the individual legally uses a lawful product off the employer's premises during nonworking hours.<br>(3) Subsection (2) does not apply to:<br>(a) Use of a lawful product, including the use of marijuana for a debilitating medical condition as defined in 50-46-302, that:<br>(i) Affects in any manner an individual's ability to perform job-related employment responsibilities or the safety of other employees; or<br>(ii) Conflicts with a bona fide occupational qualification that is reasonably related to the individual's employment;<br>(b) An individual who, on a personal basis, has a professional service contract with an employer and the unique nature of the services provided authorizes the employer, as part of the service contract, to limit the use of certain products; or<br>(c) An employer that is a nonprofit organization that, as one of its primary purposes or objectives, discourages the use of one or more lawful products by the general public.	20%

		employers where one of their primary purposes is to discourages the use of one or more lawful products by the general public.	(4) An employer does not violate this section if the employer takes action based on the belief that the employer's actions are permissible under an established substance abuse or alcohol program or policy, professional contract, or collective bargaining agreement.<br>(5) An employer may offer, impose, or have in effect a health, disability, or life insurance policy that makes distinctions between employees for the type or price of coverage based on the employees' use of a product if:<br>(a) Differential rates assessed against employees reflect actuarially justified differences in providing employee benefits;<br>(b) The employer provides an employee with written notice delineating the differential rates used by the employer's insurance carriers; and<br>(c) The distinctions in the type or price of coverage are not used to expand, limit, or curtail the rights or liabilities of a party in a civil cause of action.	
North Dakota	N.D. CENT. CODE §§ 14-02.4-01 et seq. (“State policy against discrimination ”)<br><br>1993	Does not apply if there are essential business related interests.	It is the policy of this state to prohibit discrimination on the basis of race, color, religion, sex, national origin, age, the presence of any mental or physical disability, status with regard to marriage or public assistance, or participation in lawful activity off the employer's premises during nonworking hours which is not in direct conflict with the essential business-related interests of the employer; to prevent and eliminate discrimination in employment relations, public accommodations, housing, state and local government services, and credit transactions; and to deter those who aid, abet, or induce discrimination or coerce others to discriminate.	17%
Kentucky*	KY REV. STAT. ANN. § 344.040 (“Unlawful discrimination by employers; difference in health plan contribution rates for smokers and nonsmokers and benefits for smoking cessation program participants excepted”)<br><br>1994	All Employees	(1) It is an unlawful practice for an employer:<br>(a) To fail or refuse to hire, or to discharge any individual, or otherwise to discriminate against an individual with respect to compensation, terms, conditions, or privileges of employment, because of the individual's race, color, religion, national origin, sex, age forty (40) and over, because the person is a qualified individual with a disability, or because the individual is a smoker or nonsmoker, as long as the person complies with any workplace policy concerning smoking;<br>(b) To limit, segregate, or classify employees in any way which would deprive or tend to deprive an individual of employment opportunities or otherwise adversely affect status as an employee, because of the individual's race, color, religion, national origin, sex, or age forty (40) and over, because the person is a qualified individual with a disability, or because the individual is a smoker or nonsmoker, as long as the person complies with any workplace policy concerning smoking; or<br>(c) To require as a condition of employment that any employee or applicant for employment abstain from smoking or using tobacco products outside the course of employment, as long as the person complies with any workplace policy concerning smoking.<br>(2) (a) A difference in employee contribution rates for smokers and nonsmokers in relation to an employer-sponsored health plan shall not be deemed to be an unlawful practice in violation of this section.<br>(b) The offering of incentives or benefits offered by an employer to employees who participate in a smoking cessation program shall not be deemed to be an unlawful practice in violation of this section.	29%
Mississippi	MISS. CODE ANN. § 71-7-33 (“Tobacco products”)<br><br>1991	All employees	It shall be unlawful for any public or private employer to require as a condition of employment that any employee or applicant for employment abstain from smoking or using tobacco products during nonworking hours, provided that the individual complies with applicable laws or policies regulating smoking on the premises of the employer during working hours.	26%

California	CA LABOR CODE § 96(k) (“Assignment of claims”) & 98.6 (“Discharge or discrimination, retaliation, or adverse action against employee or applicant for conduct delineated in this chapter or because employee or applicant has filed complaint or claim, instituted or caused to be instituted any proceeding under or relating to his or her rights or testified relating to the same on behalf of that person or another; reinstatement and reimbursement; penalties; employment entitlement for applicant; severability; applicability”)<br><br>2003	All Employees	Smokers may not be fired as this constitutes: “discharge from employment for lawful conduct occurring during nonworking hours away from the employer’s premises.”	15%

Connecticut	CT GEN. STAT. ANN. § 31-40s ("Smoking or use of tobacco products outside of the workplace") 1991	All Employees except firefighters and police officers. Non-profits whose primary purpose is to discourage use of tobacco products by the general public do not need to adhere this statute.	31-40s. (a) No employer or agent of any employer shall require, as a condition of employment, that any employee or prospective employee refrain from smoking or using tobacco products outside the course of his employment, or otherwise discriminate against any individual with respect to compensation, terms, conditions or privileges of employment for smoking or using tobacco products outside the course of his employment, provided any nonprofit organization or corporation whose primary purpose is to discourage use of tobacco products by the general public shall be exempt from the provisions of this section.<br>(b) Nothing contained in this section shall be construed to affect (1) the provisions of section 31-40q, (2) municipal hiring practices involving paid firefighters and paid police officers, and (3) any collective bargaining agreement between a municipality and paid firefighters or paid police officers.	18%
Rhode Island	R.I. GEN. LAWS § 23-20.10-14 ("Prohibited condition of employment--Smoking by employees outside course of employment")<br><br>2004	Any employer that is a nonprofit organization which as one of its primary purposes or objectives discourages the use of tobacco products by the general public.	(a) No employer or agent of any employer shall require, as a condition of employment, that any employee or prospective employee refrain from smoking or using tobacco products outside the course of his or her employment, or otherwise discriminate against any individual with respect to his or her compensation, terms, conditions or privileges of employment for smoking or using tobacco products outside the course of his or her employment. Provided, however, that the following employers shall be exempt from the provisions of this section: Any employer that is a nonprofit organization which as one of its primary purposes or objectives discourages the use of tobacco products by the general public.<br>(b) In any civil action alleging a violation of this section, the court may;<br>(1) Award up to three (3) times the actual damages to a prevailing employee or prospective employee;<br>(2) Award court costs to a prevailing employee or prospective employee;<br>(3) Afford injunctive relief against any employer who commits or proposes to commit a violation of this chapter.<br><br>(c) Nothing contained in this chapter shall be construed to affect any other provisions of this title.	20%
Indiana*	IND. CODE § 22-5-4-1 **("**Condition of employment; discrimination; financial incentives")<br><br>1991	All employees	"discriminate against an employee with respect to: the employee's compensation and benefits"<br><br>"based on the employee's use of; tobacco products outside the course of the employee's or prospective employee's employment."<br><br>Sec. 1. (a) Except as provided in subsection (b), an employer may not:<br>(1) Require, as a condition of employment, an employee or prospective employee to refrain from using; or<br>(2) Discriminate against an employee with respect to:<br>(A) The employee's compensation and benefits; or<br>(B) Terms and conditions of employment;<br>based on the employee's use of;<br>tobacco products outside the course of the employee's or prospective employee's employment.<br>(b) An employer may implement financial incentives:<br>(1) Intended to reduce tobacco use; and<br>(2) Related to employee health benefits provided by the employer.	25%
